# Low‐Temperature Charging and Aging Mechanisms of Si/C Composite Anodes in Li‐Ion Batteries: An Operando Neutron Scattering Study

**DOI:** 10.1002/cssc.201903139

**Published:** 2019-12-27

**Authors:** Karsten Richter, Thomas Waldmann, Neelima Paul, Nicola Jobst, Rares‐George Scurtu, Michael Hofmann, Ralph Gilles, Margret Wohlfahrt‐Mehrens

**Affiliations:** ^1^ Zentrum für Sonnenenergie- und Wasserstoff-Forschung, Baden-Württemberg Helmholtzstrasse 8 89081 Ulm Germany; ^2^ Heinz Maier-Leibnitz Zentrum Technische Universität München Lichtenbergstr. 1 85748 Garching Germany

**Keywords:** batteries, lithium, mechanism, operando study, silicon

## Abstract

The addition of Si compounds to graphite anodes has become an attractive way of increasing the practical specific energies in Li‐ion cells. Previous studies involving Si/C anodes lacked direct insight into the processes occurring in full cells during low‐temperature operation. In this study, a powerful combination of operando neutron diffraction, electrochemical tests, and post‐mortem analysis is used for the investigation of Li‐ion cells. 18650‐type cylindrical cells in two different aging states are investigated by operando neutron diffraction. The experiments reveal deep insights and important trends in low‐temperature charging mechanisms involving intercalation, alloying, Li metal deposition, and relaxation processes as a function of charging C‐rates and temperatures. Additionally, the main aging mechanism caused by long‐term cycling and interesting synergistic effects of Si and graphite are elucidated.

## Graphical abstract

**Charge, relax, discharge**! Li distribution in 18650‐type Li‐ion batteries with Si/C composite anode is investigated by operando neutron scattering, which allows the time evolution of the LiC_6_ and LiC_12_ reflections to be measured and, combined with electrochemical tests and post‐mortem analysis, reveals details on the charging, relaxation, and aging mechanisms.

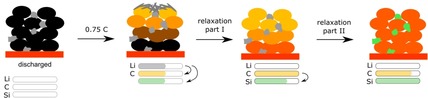

## Introduction

Extensive burning of fossil fuels has led humanity into a global climate crisis.^1^ One key to reducing CO_2_ emissions and decelerating global warming is the development of energy storage materials with high energy densities/specific energies in combination with renewable energy sources. The utilized materials have to be sustainable, efficient, cheap, and reliable. Li‐ion batteries have demonstrated a key role in a variety of mobile applications. However, their specific energies need to be further improved, for example, to achieve sufficient driving ranges in electric cars. To increase the cycle life of batteries in applications, the aging mechanisms responsible for their performance loss need to be well understood. This would allow mitigation of their degradation and increased sustainability through a knowledge‐based approach. If batteries have a longer life, fewer batteries have to be produced and replaced or recycled and less resources have to be mined (e.g., Co for cathode active materials).

Si is a prominent candidate for negative electrode (anode) materials,^2^, ^3^, ^4^, ^5^, ^6^, ^7^, ^8^, ^9^, ^10^ owing to its abundance in the earth's crust^11^ and its high theoretical capacity.^5^ However, owing to its high volume expansion (up to 300 %), the resulting particle cracks, and its reaction with the electrolyte,^5^ one strategy is to combine the high gravimetric capacity of Si compounds with graphite.^4^, ^6^, ^7^, ^9^, ^10^ For example, the cells tested in this work contain 3.5 wt % Si,^9^, ^10^ leading to a specific energy that is comparable with that of other state‐of‐the‐art cells.^12^ If 3.5 wt % Si is added and the anode coating thickness is kept constant at approximately 52 μm, the specific capacity increases from approximately 135 Wh kg^−1^ to 180 Wh kg^−1^ on a cell level. Conversely, the addition of 3.5 wt % Si results in a decrease in the anode coating thickness from approximately 80 μm (pure graphite) to 52 μm (graphite+Si compound) if the areal capacity is kept constant.

For room‐temperature alloying of Si, onset potentials from approximately 1 V to 0.15 V have been reported, depending on the particle size, electrolyte, and active material composition.^4^, ^5^, ^7^ Pure graphite anodes show four lithiation stages, starting from approximately 0.2 to 0 V versus Li/Li^+^.^13^ Below 0 V versus Li/Li^+^, Li metal deposition on the anodes surface becomes thermodynamically possible.^14^ Li deposition is a severe side reaction,^15^, ^16^ which is caused by overpotentials favored by charging at low temperatures, high current densities, and high lithiation states.^17^, ^18^, ^19^ Li metal deposition on anodes leads to fast aging by reaction with the electrolyte.^15^, ^16^ Furthermore, Li deposition can cause safety risks leading to thermal runaway owing to exothermic reactions,^20^ and the dendritic structures of deposited Li can cause internal short circuits.^16^

Deposited Li is known to re‐intercalate into graphite anodes in rest periods after charging.^20^, ^21^, ^22^, ^23^ In the 1980s, several groups found hints that Li atoms evaporated onto a highly oriented pyrolytic graphite (HOPG) single crystal readily intercalate into the graphite lattice.^24^, ^25^ The re‐intercalation of deposited Li into graphite anodes of cylindrical Li‐ion cells at −20 °C^21^ and at −2 °C^22^ was recently investigated by neutron diffraction experiments. In these experiments, no diffraction peaks were observed for pure Li; however, re‐intercalation of Li was indirectly observed by monitoring the increase/decrease of the LiC_6_ and LiC_12_ phases.^21^, ^22^ Low temperatures are typically chosen to slow down the processes and thereby increase the probability of observing Li deposition.^21^, ^22^ Li deposition is also a relevant mechanism for fast charging above room temperature.^26^

It is not surprising that the mixed potential of Li_*x*_C_6_ and Li causes changes in the cell voltage. Uhlmann et al. were first to report that re‐intercalation of deposited Li in half cells is indicated by a voltage plateau during relaxation after charging.^23^ The occurrence of this voltage plateau was later corroborated by von Lüders et al. by using neutron diffraction data for experiments with a Li‐ion full cell containing a graphite anode.^22^ Recently, we confirmed the relationship between the plateau in the voltage curve with a decrease of deposited Li by post‐mortem analysis and glow discharge optical emission spectroscopy (GD‐OES).^20^ Yao et al. recently investigated Si/C composite anodes (15 % Si) in half cells by using energy‐dispersive XRD.^4^ For coin half cells, they found that nanometer‐sized Si was alloying before the lithiation of graphite at low C‐rates.^4^

**Figure 1:**
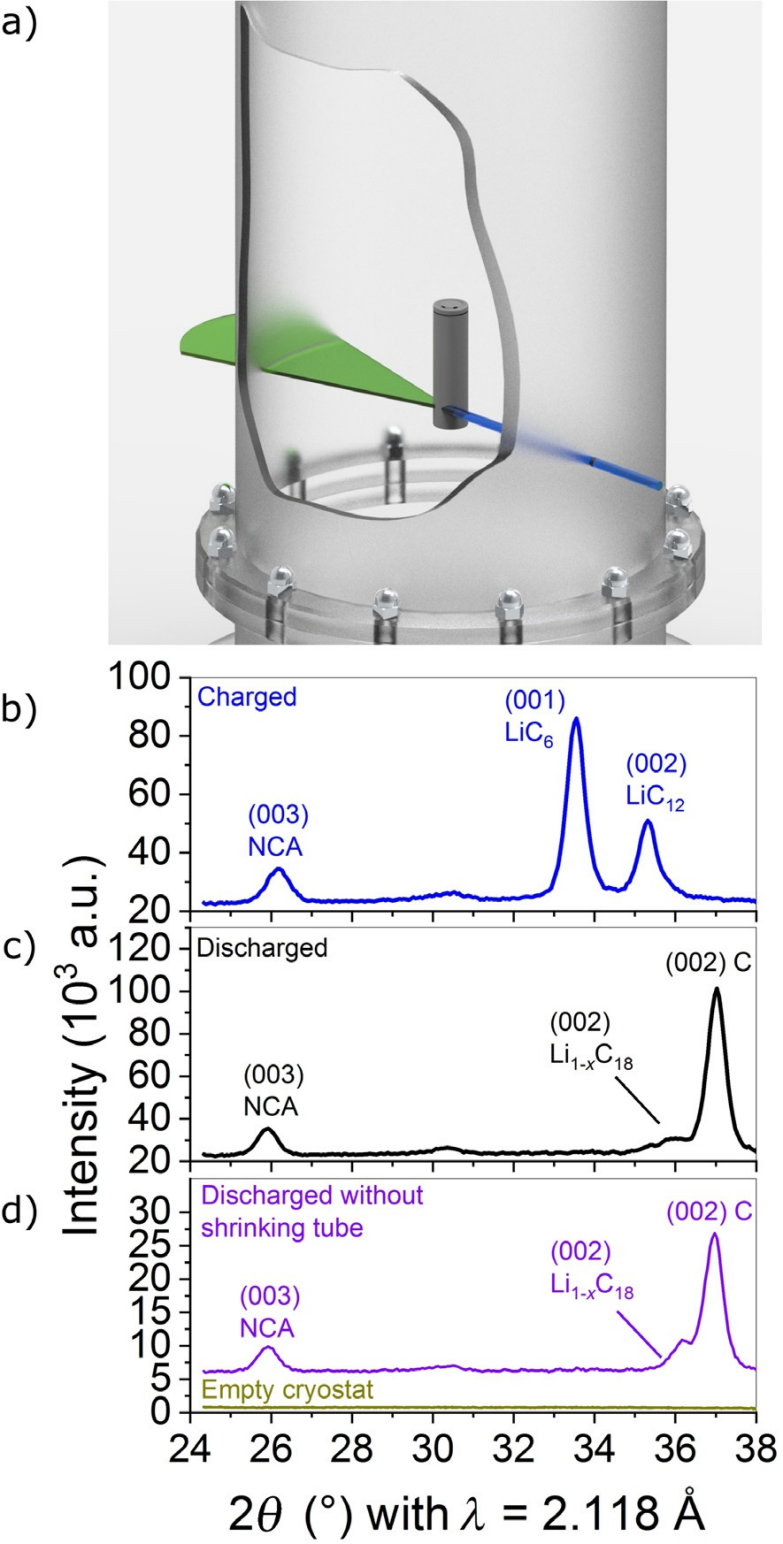
a) Schematic diagram of the investigated 18650‐type cell, with the positioning of the incoming neutron beam (blue) and the diffracted neutrons (green). The cell was positioned inside a He‐filled cryostat to maintain a constant operating temperature. b–d)) Excerpt of the recorded diffractogram measured using the fresh cell in b) a charged state (blue); c) discharged state (back); and d) in the discharged state and without the shrinking tube (purple) and the empty cryostat (green–brown).

However, there is a dearth of knowledge on the charging and aging mechanisms of Si/C composite anodes containing micrometer‐sized particles in full cells, even though these are key components that need to be understood to improve low‐temperature aging, fast‐charging capability, and battery cycle life. In particular, there is a lack of knowledge about Li deposition on Si/C composite anodes. Therefore, in this study we used operando neutron diffraction as a powerful method to obtain deeper insights into the processes that occur in Si/C anodes during the operation of Li‐ion cells. In our experiments, incident neutrons were scattered from the electrodes inside a cylindrical Li‐ion cell with a Si/C composite anode (Figure 1 a; particle diameter≈10 μm) during charging and subsequent relaxation. This allowed simultaneous investigation of the LiC_12_ and LiC_6_ phase fractions, their changes with time, and electrochemical data. The influence of temperature, charging C‐rate, and state‐of‐health (SOH) on the relaxation processes inside the active material of Si/C composite anodes was investigated in detail to unravel the effects of Si compounds on the charging and aging mechanisms. Post‐mortem analysis of the cells was conducted as a complimentary method for analysis of the aging mechanisms.

## Results

### Identification of relevant reflections in Neutron diffractograms

As shown in Figure 1 a, the incident neutrons were scattered from the Si/C anode and the Li_*x*_Ni_0.86_Co_0.10_Al_0.04_O_2_ (NCA) cathode of the 18650‐type cell. The neutron diffraction data in the discharged and charged state are shown in Figure 1 b, c. For the discharged state, the angular position of the reflexes were 2*θ* of NCA (003)=25.923(7)°, 2*θ* of C (002)=37.025(6)° and for the charged state, 2*θ* of NCA (003)=26.210(4)°, 2*θ* of LiC_6_ (001)=33.553(3)°, 2*θ* of LiC_12_ (002)=35.3179°.

During electrochemical operation, the diffraction angle of the NCA reflex changed owing to the oxygen repulsion, which is typical for layered oxides.^27^ However, in the break after charging, neither its intensity nor its position changed (see Supporting Information, Figures S2 and S3). All other reflexes were attributed to the active materials, casing, and current collectors, except one low intensity peak at 2*θ*=30.5°. This reflex was not observed in a measurement of the empty cryostat (Figure 1 d) and did not change with charging. Therefore, it is likely to be caused by an inactive material.

The Si compound was not visible either in the neutron diffraction data or in the XRD measurements of the anode taken from the disassembled cell. This was expected because Si can become amorphous during electrochemical treatment.^8^ However, as shown below, the effects of the Si compound can be indirectly observed through analysis of the LiC_6_ and LiC_12_ reflexes, similar to previous indirect observations of Li deposition.^21^, ^22^

### Relaxation mechanism after charging in fresh cells

**Figure 2:**
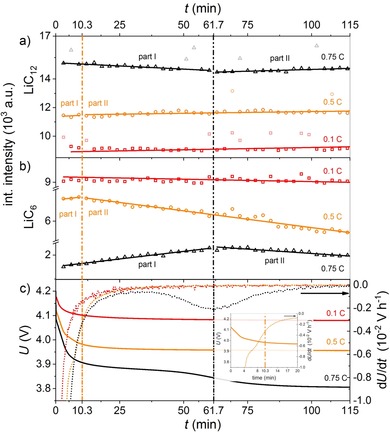
Evolution of the integral intensities of the a) LiC_12_ and b) LiC_6_ reflections after charging at 0.1 C, 0.5 C, and 0.75 C and c) respective voltage (left axis) and differential voltage curves (right axis) at −21±2 °C. The transitions between the different relaxation phases (part I&II) are marked by the vertical dashed lines. Outliers are displayed in faded colors and were not taken into account for the fitting. The inset in (c) shows a magnification for better visibility of the curve for 0.5 C charging (see also Figure S1).

To obtain insights into the charging mechanism of a fresh cell, operando neutron scattering experiments were performed with a fresh cell at −21±2 °C for different C‐rates. The evolution of the integrated intensities for the LiC_12_ and LiC_6_ reflexes during relaxation after the charging are shown in Figure 2 a, b. The voltage relaxation and differential voltage curves that were recorded simultaneously are shown in Figure 2 c.

For charging at rates of 0.1, 0.5, and 0.75 C, the capacities with respect to charging at 1 C (2.5 Ah) at room temperature were 81.4 % (2.03 Ah), 68.7 % (1.71 Ah), and 46.6 % (1.16 Ah), respectively. This reversible reduction of the charge capacity with increasing C‐rate is typical for Li‐ion batteries and is not an aging effect.

**Figure 3:**
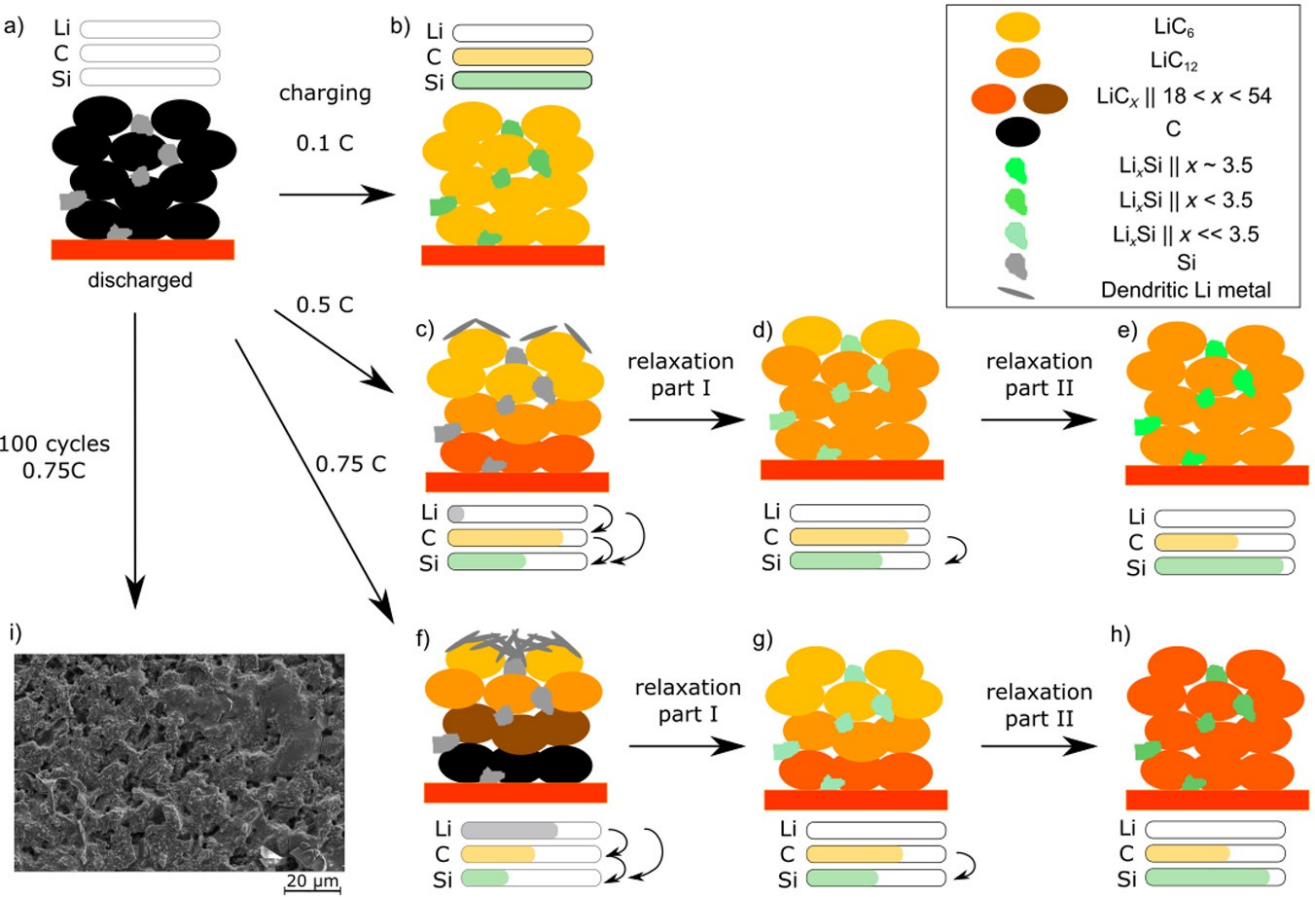
a) Schematic diagram of the charging, relaxation, and Li redistribution process in Si/C composite anodes at −21±2 °C and different charging C‐rates. a) Discharged cell; b) charging at 0.1 C; c–e) 0.5 C; f–h) 0.75 C. i) SEM of the anode surface after continuous 0.75 C cycling at −21 °C. The amounts of Li are intended to show the observed trend rather than quantitative values.

There were no significant changes in the integral intensities of the LiC_6_ and LiC_12_ phase in the neutron diffraction data after 0.1 C charging (Figure 2 a,b). The lithiation of the anode from the electrode surface to the current collector was expected to be homogeneous for 0.1 C charging because polarization effects are minor^28^, ^29^ (see Figure 3 a, b for model). The voltage and the differential voltage relaxation curves (Figure 2 c) did not contain a voltage plateau for 0.1 C charging. Therefore, no Li deposition was expected for this case.^20^, ^22^, ^23^

An increasing propensity for Li deposition on the anode was expected with increasing charging C‐rate. Indeed, Li was deposited on the Si/C anode during 0.5 C charging, as indicated by the plateau in the voltage curve (Figure 2 c). The evolution of the integrated intensity after charging at 0.5 C could be divided into two parts, which, to our knowledge, has not been observed before: part I from 0 to 10.3 min and part II from 10.3 min to 115 min after charging. For LiC_6_, both parts fit well to linear functions (part I: 11.19±4.72 min^−1^, part II: −13.71±0.54 min^−1^). Most interestingly, the change from part I to part II happened at approximately the end of the plateau in the voltage curve (marked by a dashed line) and at the turning point of the differential voltage curve. The shape of the voltage curve and the observed increase of the LiC_6_ in part I of the relaxation was consistent with re‐intercalation of Li metal into graphite according to Equation (1 a) a, b):

(1 a) $$\mathrm{Li}\to \mathrm{Li}{}^{+}+e{}^{-}$$

(1 b) $$\mathrm{Li}{}^{+}+e{}^{-}+\mathrm{LiC}{}_{12}\to 2\;\mathrm{LiC}{}_{6}$$

The reason was that the amount of LiC_6_ increased after the end of the charge, that is, without current flow. Therefore, the LiC_6_ intensity data in part I during the relaxation after 0.5 C charging was consistent with the data reported by other researchers, who have observed re‐intercalation of deposited Li into graphite electrodes (without Si compound) by neutron diffraction.^21^, ^22^ In part I, a partial transfer of Li to the Si compound according to Equation (1c) cannot be ruled out from the present data set.

(1 c) $$\mathrm{Li}{}^{+}+e{}^{-}+\mathrm{Li}{}_{x}\mathrm{Si}{}_{y}\to \mathrm{Li}{}_{x+1}\mathrm{Si}{}_{y}$$

For the LiC_12_ intensity, no decrease was observed in part I after charging with 0.5 C. However, the time window for the detection of the LiC_12_ could be too short, that is, only three data points are most likely not enough for a linear fit. Part II of the relaxation showed a linear decrease for the LiC_6_, which has not been reported in previous papers. Because of the lack of driving force, this decrease cannot be assigned the reverse reaction of [Eq. (1 b)], that is, the formation of Li metal. Furthermore, there was no current flow in this part of the experiment and therefore no electrochemical de‐intercalation of Li from graphite. However, it is likely that Li from LiC_6_ was transferred to the Si compound during the relaxation according to Equation (1 c) c) after de‐intercalation of Li from LiC_6_. This was also supported by the fact that this decrease of LiC_6_ amount in the relaxation after charging was not observed for pure graphite anodes that do not contain a Si compound.^21^, ^22^

Similar relaxation effects have been reported for blend cathodes.^30^, ^31^, ^32^ Klein et al. observed relaxation processes by in situ XRD after moving the system away from the equilibrium situation by pulse power discharge tests.^30^ In their experiments, Li^+^ ions were preferentially transferred into the LiMn_1.9_Al_0.1_O_4_ spinel (LMO) material owing to its faster kinetics.^30^ In the relaxation phase after the pulse, the Li^+^ ions were redistributed to the LiFe_0.3_Mn_0.7_PO_4_ (LFMP) material.^30^ This was similar in our experiment, in which graphite seemed to show the faster kinetics compared with the Si compound. However, this effect very likely depends on the particle size distribution.

During charging of the Si/C composite, the graphite particles are most likely preferentially lithiated before the Si compound (see Figure 3 a, c–e for model). Thermodynamically, Si should be lithiated before graphite.^4^ However, charging at a high rate of 0.5 C moves the system away from the equilibrium situation. In contrast, charging at a low rate of 0.1 C is closer to the equilibrium situation and therefore no relaxation effects were observable (see above).

A similar time evolution of the LiC_6_ integrated intensity was observed during the rest period after charging at a rate of 0.75 C compared with that at 0.5 C. In contrast to charging at 0.5 C, we observed a decrease in the LiC_12_ integrated intensity in part I, which was in accordance with the data reported by Zinth et al.^21^ Again, for 0.75 C charging we observed a coincidence of the turning point of the differential voltage curve and a change from part I to part II of the relaxation in the neutron diffraction data. However, part I (re‐intercalation of deposited Li) for 0.75 C was longer compared with that of 0.5 C charging; the reason was stronger polarization of the anode at higher C‐rates, which was most likely caused by a higher amount of deposited Li (see Figure 3 a, f–h for model).

The slopes in part II for the LiC_6_ integrated intensities were also different for 0.5 C and 0.75 C charging (−7.12±0.83 min^−1^). However, a direct comparison was difficult because it is likely that the re‐intercalation/alloying of deposited Li metal is not completed and an overlap of the Equations (1 a–c) might be occurring.

An SEM image of an anode after disassembly of a cell cycled 100 times with a charging C‐rate of 0.75 C at −21 °C is shown in Figure 3 i. The graphite particles were covered by a Li film, which was in agreement with the neutron diffraction and electrochemical data obtained under similar conditions (−21 °C, 0.75 C charging). The deposited Li was not dendritic, which has previously been observed for another cell type,^33^ most likely because of the pressure in the cylindrical cells and reaction with electrolyte.

From a thermodynamic point of view at room temperature, the onset of Si alloying is before that of graphite lithiation.^4^, ^5^, ^7^ For Li diffusion in lithiated graphite and in Si, the barriers are in the range of 0.2–0.3 eV^34^, ^35^ and 0.62 eV,^36^, ^37^ respectively. However, with increasing currents and lower temperatures, kinetics in the form of activation barriers have to be considered. This substantiates the kinetically controlled primary lithiation of graphite compared with the Si compound in this case (diameter≈10 μm). In the case of Si nanoparticles, for which the paths for solid diffusion are lower by several orders of magnitude, Si was observed to be lithiated before graphite.^4^, ^7^

### Post‐mortem analysis of main aging mechanism at 25 °C

**Figure 4:**
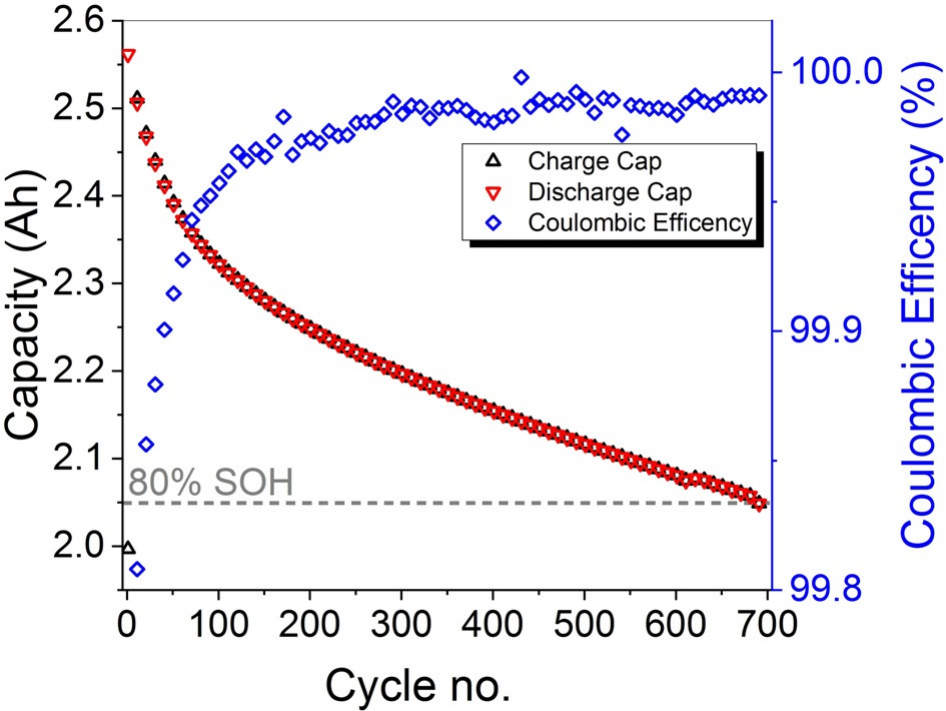
Charge (black triangles upwards), discharge capacity (red triangles downwards) and coulombic efficiency (blue rhombohedra, right *y*‐axis) vs. cycle number of the aged cell (25 °C, 1 C). 80 % SOH is marked with the grey dashed horizontal line. For better visibility, only every 10th data point is displayed.

The cycling stability of the investigated 18650‐type cells during cycling at 25 °C and 1 C is shown in Figure 4. The strong initial capacity loss was in accordance with the low coulombic efficiency within the first 100 cycles. Therefore, the capacity loss over the first 100 cycles (0.239 Ah) was nearly as high as the cumulative capacity loss during the next 600 cycles (0.242 Ah). The absence of a sudden capacity drop observed for other cell types^38^ indicated that the main aging mechanism does not change drastically during cycling. In contrast, the capacity fade decelerated, indicating that the amount of cyclable Li decreased in the aged cell whereas the usable active materials stayed relatively constant.^39^ The decelerated capacity fade was consistent with the increase in coulombic efficiency, indicating fewer side reactions with increasing cycle number.

**Figure 5:**
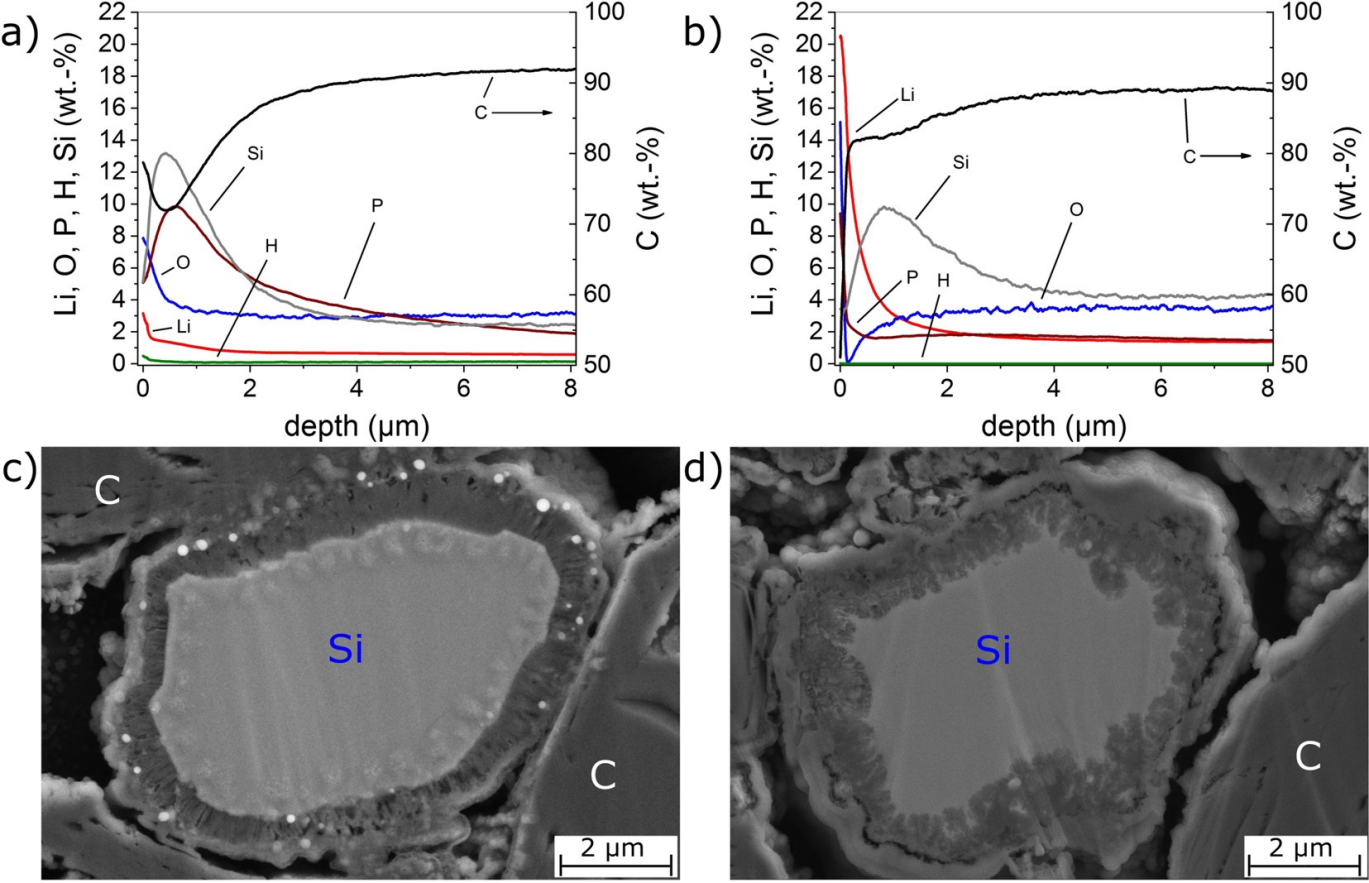
Post‐mortem analysis of the fresh and aged cell with Si/C composite anode cycled 700 times at 25 °C at a rate of 1 C. a, b) GD‐OES depth profiles of a) fresh and b) aged Si/C electrode. c, d) SEM images of the anode cross‐section of c) fresh and d) aged Si/C electrode.

The post‐mortem analysis results after disassembly of a fresh cell and a cell cycled at 25 °C are shown in Figure 5. A different Si distribution was detected for the aged cell (Figure 5 b) compared with the fresh cell (Figure 5 a). A lower maximum and a broader distribution was observed for the Si distribution of the aged cell, which indicated that the formed film extended more into the depth of the anode compared with the anode from the fresh cell. These results for the cell cycled at 25 °C were consistent with depth profiling results for the same cell type cycled at 0 °C and 45 °C reported previously.^10^ The most likely formed Li silicates consume cyclable Li,^10^ which was consistent with the decelerated capacity fade curve in Figure 4. The loss of cyclable Li has also been observed for other cells without Si compound in the anode.^39^, ^40^, ^41^, ^42^, ^43^, ^44^ Therefore, a part of the capacity loss in the cells was likely to originate from the growth of a solid electrolyte interphase (SEI) layer on the graphite particles in addition to the aging effect of the Si compound; however, a large part of the film growth and the low coulombic efficiency can most likely be attributed to the formation of Li silicates.

The GD‐OES depth profile of the aged cell (Figure 5 b) shows high Li and O values at the anode surface, representing additional electrolyte consumption and SEI formation; furthermore, the aged cell shows no P maxima at the electrode surface, indicating the consumption of conductive salt and the dissolution of P‐containing species form the SEI.

Aging of the Si compound was also visible at the border of the particles (compare Figure 5 c for fresh cell and Figure 5 d for aged cell). A detailed comparison of the images of the particle cross‐sections supported consecutive film formation predominantly on the Si particles. Additionally, the surface film on the particles seemed to be de‐contacted, and the particle itself showed no sharply separated but more frayed interface to the formed film.

### Effects of temperature and aging on the intercalation mechanism in fresh and aged cells

**Figure 6:**
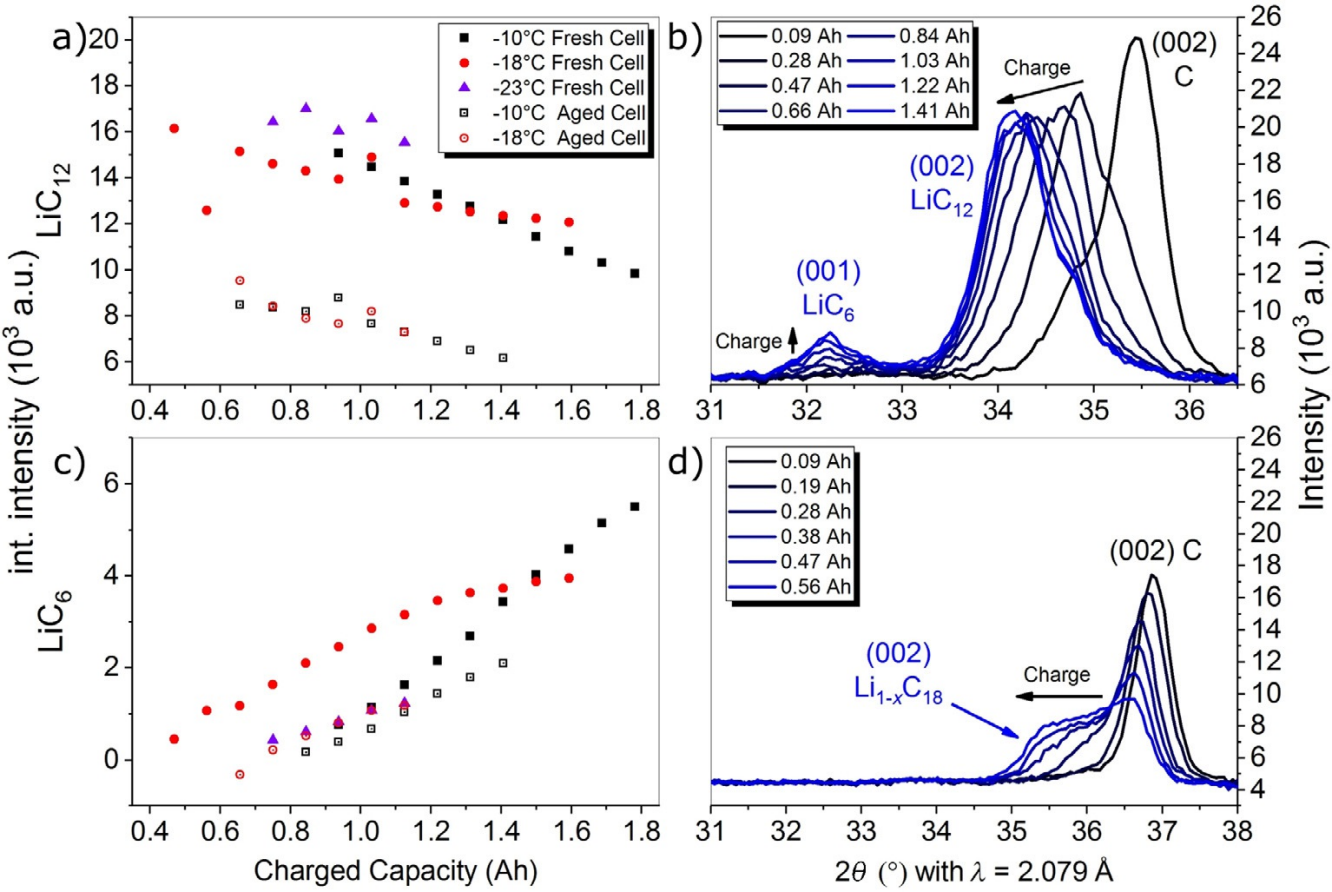
Operando neutron diffraction data recorded during charging. a) Integrated intensities of the LiC_12_ reflection during charging with 0.75 C at −10 °C (black squares), −18 °C (red circles) and −23 °C (purple triangles) at 100 % SOH (filled data points) and 80 % SOH (empty data points) and c) the LiC_6_ integrated intensities. b) Excerpt of diffraction data series recorded every 3 min during the charging at −23 °C and 100 % SOH. d) Excerpt of diffraction data series recorded every 3 min during the charging at −23 °C and 80 % SOH.

Insights into the charging mechanism as a function of temperature is a major key for understanding both the main aging mechanism and fast‐charging capability of a cell. The neutron diffraction experiments during charging at a fixed rate of 0.75 C in the range of −23 °C to −10 °C is shown in Figure 6. In these experiments, a fresh (discharge capacity at 25 °C: 2.56 Ah, 100 % SOH) was compared with an aged cell (aging conditions: 700 cycles at 1 C/25 °C, discharge capacity at 25 °C: 2.04 Ah, 79.6 % SOH).

For charging of the fresh cell at −10 °C (Figure 6 a and c, black squares), a mostly homogenous lithiation of the anode was expected as the LiC_12_ integrated intensity linearly decreased and the LiC_6_ integrated intensities linearly increased, corresponding to Equation (1 b). At −10 °C, the slope of the LiC_6_ phase seemed to flatten for the last data point, which weakly indicated the starting point for Li metal deposition during galvanostatic charging.

For charging of the fresh cell at −18 °C, the curve shape clearly started to deviate from the results at −10 °C. The LiC_6_ phase contribution appeared at a lower charged capacity (0.44 Ah for −18 °C compared with 0.94 Ah for −10 °C). This indicated an inhomogeneous phase distribution over the electrode depth because the ratio between LiC_6_ and LiC_12_ was stronger. From simulations, more of the LiC_6_ was expected to be located at the anode surface (near the separator).^45^ This was in accordance with measurements^20^, ^33^, ^42^, ^46^ and simulations^17^ that have shown that Li deposition (favored by high lithiation degrees)^18^, ^47^ is also mostly located on the anode surface.

At a charge capacity of approximately 1.2 Ah, the LiC_6_ and LiC_12_ integrated intensities flattened, even though the galvanostatic charging delivers a constant flux of Li^+^ ions. This is an indication of Li metal deposition on the Si/C anode. Therefore, we assigned the start of Li deposition for the fresh cell between −10 °C and −18 °C at a charging rate of 0.75 C. For charging at −23 °C, only small amounts of the LiC_6_ phase was observed, which was in accordance with the above results.

The LiC_12_ and LiC_6_ integrated intensities for the aged cell were different from those of the fresh cell. The phase contributions of both LiC_12_ and LiC_6_ were lower than those for the fresh cell. No LiC_6_ contribution was observed for the aged cell charged at −23 °C, which was consistent with the results from the post‐mortem analysis and with the shape of the capacity fade curve. However, for both the fresh and the aged cell, the neutron diffraction data series(Figure 6 b, d) show contributions from LiC_*x*_ (*x*≥18), underlining the importance of Equation (2):

(2) $$\mathrm{LiC}{}_{6}+\mathrm{LiC}{}_{18}\to 2\;\mathrm{LiC}{}_{12}$$

**Figure 7:**
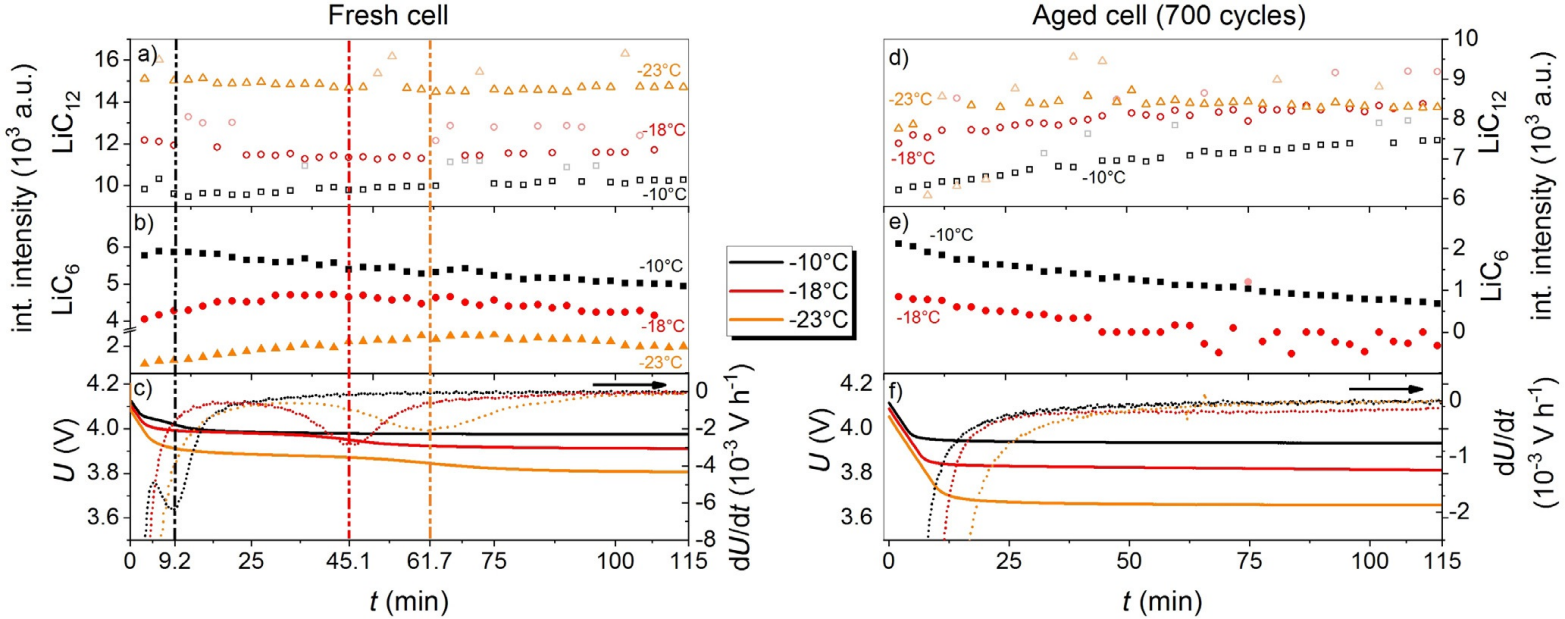
a, d) Integrated intensities during the relaxation time of LiC_12_ after charging with 0.75 C at −10 °C (black squares), −18 °C (red circles), and −23 °C (orange triangles) at a) 100 % SOH and d) at 80 % SOH. b, e) Integrated intensities during relaxation time of LiC_6_ after charging with 0.75 C at −10 °C (black squares), −18 °C (red circles), and −23 °C (orange triangles) at b) 100 % SOH and e) at 80 % SOH. c, f) Relaxation voltage profiles (left axes, solid lines) and differential voltage (right axes, dotted lines) at −10 °C (black), −18 °C (red), and −23 °C (orange) at c) 100 % SOH and f) 80 % SOH. Outliers are displayed in faded colors.

The integrated intensities, voltage curves, and differential voltages during the relaxation after charging at a rate of 0.75 C between −10 °C and −23 °C are shown in Figure 7. The integrated intensities of LiC_12_ and LiC_6_ during relaxation for the fresh cell are shown in Figure 7 a, b. Similar to that in Figure 2, the relaxation was divided into two parts. Again, the transitions observed in the neutron diffraction data were coincided with the electrochemical data, that is, the transitions occurred at the inflection points of the voltage curves (see vertical dashed lines in Figure 7 a–c). This was also consistent with the data in Figure 6 recorded during charging.

The time at which the transition from part I to part II occurs is temperature dependent. After charging at −10 °C, −18 °C, and −23 °C, the transition happens after 9.2 min, 45.1 min, and 61.7 min, respectively. The longer relaxation times at lower temperatures indicated more deposited Li at lower temperatures.^22^ However, lower temperatures also decrease the relaxation rate for re‐intercalation of Li into graphite.^48^ Nevertheless, the indicated trend of more Li deposition at lower temperatures was in agreement with the trend of decreasing anode potentials versus Li/Li^+^ for charging at lower temperatures.^18^, ^19^, ^49^

The integrated intensities of LiC_12_ and LiC_6_ during relaxation for the aged cell are shown in Figure 7 d,e. In contrast to the fresh cell, there was no relaxation in two parts for the aged cell. Instead, the LiC_6_ integrated intensities only decreased during relaxation, indicating only a transfer of Li from lithiated graphite to the Si compound. However, no re‐intercalation of Li into graphite was observed for the aged cell. This was in very good agreement with the voltage and differential voltage curves in Figure 7 f, which do not show plateaus or minima, respectively.

The LiC_6_ phase was not obtained during charging at −23 °C/0.75 C. For the aged cell, this charging condition resulted in only 0.60 Ah charge capacity and a comparably high voltage difference of 0.54 V during the relaxation period, indicating strong polarization effects. Consistent with the post‐mortem analysis, the consumption of cyclable Li^+^ ions provided by the cathode was not high enough for either the formation of LiC_6_ or for Li metal deposition.

## Conclusions

Simultaneous operando neutron diffraction and electrochemical assessment combined with post‐mortem analysis were used to gain valuable new insights into the charging and aging mechanisms of Si/C composite anodes (3.5 wt % Si, ≈10 μm particle size of the Si compound) of Li‐ion batteries. At −21±2 °C, the charging mechanism of a fresh cell depended on the charging C‐rate (see Figure 3 for model). In particular, slow charging (0.1 C) most likely led to simultaneous lithiation of graphite and the Si compound. No significant redistribution of Li was observed after charging. During charging at a moderate C‐rate (0.5 C), small amounts of Li metal were deposited as a parallel reaction to lithiation of graphite and alloying of the Si compound. The relaxation after charging could be divided into two parts. The transition of both parts occurred at the inflection point of the differential voltage curve. In part I of the relaxation, deposited Li re‐intercalated into graphite (and likely also into the Si compound). Most interestingly, in part II of the relaxation, Li was redistributed from lithiated graphite to the Si compound. For faster charging (0.75 C), the charging and relaxation mechanisms were similar to charging at 0.5 C; however, the Li deposition effect was stronger, that is, more Li metal was deposited on the anode surface.

**Figure 8:**
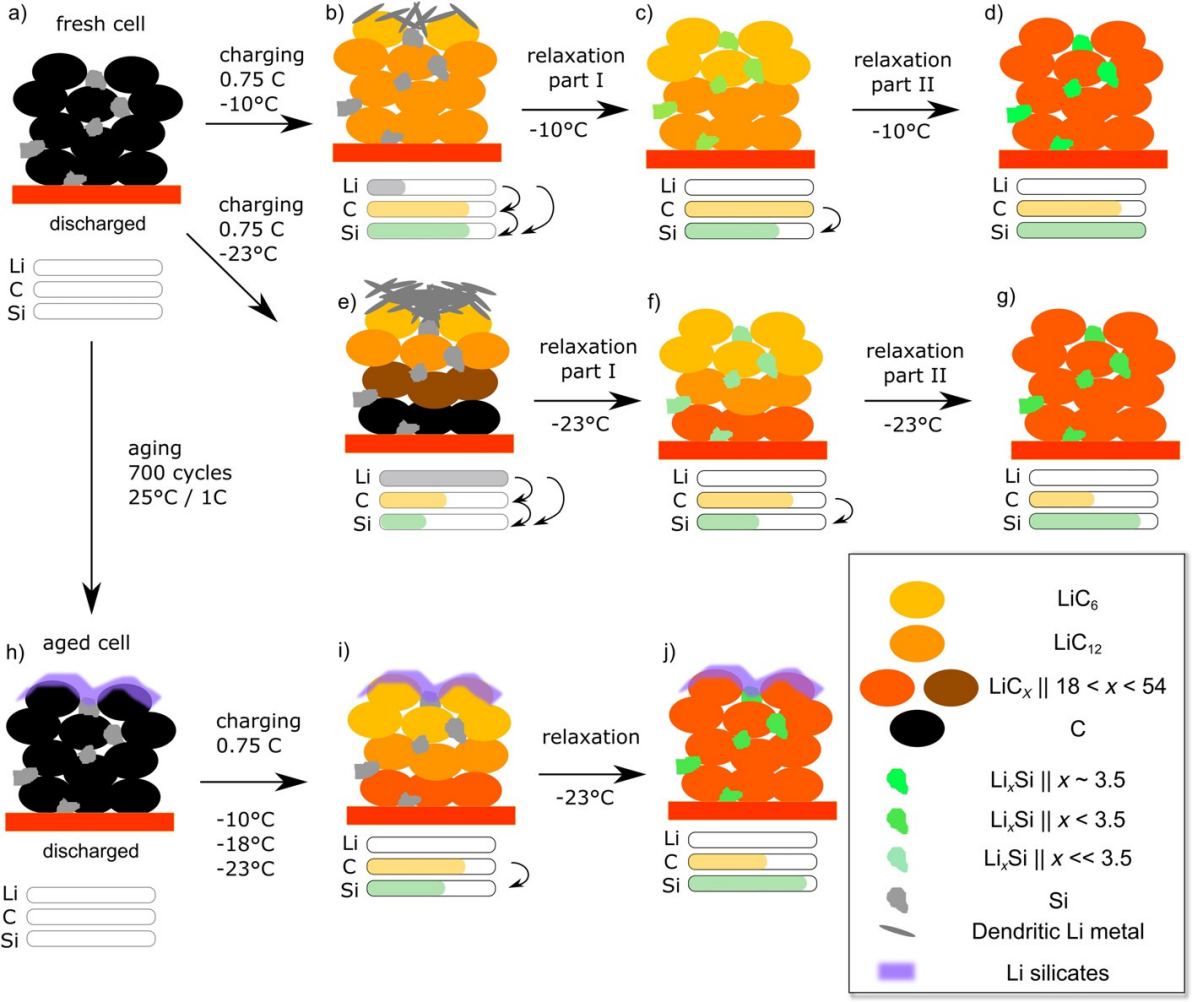
Overview of the processes that occur during low‐temperature charging of fresh and aged cells with Si/C anodes. Discharged Si/C anodes of a) fresh and h) aged cells. Charging and relaxation of the fresh cell at b–d) −10 °C/0.75 C and e–g) −23 °C/0.75 C. i) Charging and j) relaxation of the aged cell with a silicate film^10^ in which Li deposition was not observed. The amounts of Li are intended to show the observed trend rather than quantitative values.

Regarding the influence of temperature and charging at 0.75 C, we found the following trends: Li deposition on the anode occurred in the range of −10 °C to −23 °C for the fresh cells (see Figure 8 a–g for model). Lower temperatures led to longer relaxation times of part I and most likely to higher amounts of deposited Li. In contrast to the fresh cells, cells cycled at 25 °C/1 C cells did not show any evidence of Li deposition under the same conditions (see Figure 8 h–j for model). The reason for the absence of Li deposition in the aged cell was the lower amount of cyclable Li, which prevented higher lithiation states of the graphite. Fully consistent with this, in the case of charging at −23 °C and 0.75 C, neutron diffraction did not show any LiC_6_ phase contribution. The loss of cyclable Li as the main aging mechanism at 25 °C in the investigated cell type was consistent with the capacity fade curve and with post‐mortem analysis.

Moreover, our experiments showed interesting synergistic effects of the Si/C composite anodes. In the investigated cells at low temperatures, high charging C‐rates led to lithiation of the graphite before the Si compound (≈10 μm diameter) owing to kinetic limitations. Therefore, graphite acted as a kinetic buffer to store Li^+^ ions during charging of Si/C anodes. During relaxation after charging, the Li^+^ ions were transferred from graphite to the Si compound. This study showed that Si/C composite anodes behave differently to pure graphite anodes if Li deposition comes into play, that is, at low charging temperatures or under fast‐charging conditions. The results of our study will significantly enhance the understanding of the charging and aging mechanisms of Li‐ion batteries with Si/C composite anodes and suggest ways to tailor the anodes for the development of next‐generation batteries. In particular, the formation of Li silicates and the deposition of Li metal on Si/C anodes have to be prevented to enhance battery lifetime. Further studies on these topics are ongoing in our laboratories.

## Experimental Section

The investigated commercial cells have a typical capacity of 2.5 Ah and voltage range of 2.0–4.2 V. A cell mass of 48.78±0.08 g, voltage of 3.470±0.004 V, and internal resistance of 10.5±0.2 mΩ (Hioki, 1 kHz) for a batch of 30 cells showed the high quality and the suitability of the cells for further tests. Cell aging was performed by BaSyTech CTS (2.0–4.2 V) at 1 C (2.5 A) for 700 cycles at 25 °C to reach 80 % SOH. A VMP/Z electrochemical workstation (BioLogic VSP potentiostat) was used for the electrochemical operation during neutron diffraction. The charging C‐rate and temperature varied according to the experimental procedure. A discharge rate of 1 C with a 1 h relaxation time was used.

Operando neutron data were collected at the material science diffractometer STRESS‐SPEC (FRM II, Heinz Maier‐Leibnitz Zentrum, TU München). A Ge (511) monochromator was used to obtain a wavelength of 2.118 Å, determined using the NIST SRM 640d Si standard powder. The detection region was chosen to be 24°<2*θ*<38° to cover all stages of graphite (002) reflections during lithiation including the LiC_12_ (002) reflection with 2*θ*=35.317(9)° and LiC_6_ (001) reflection with 2*θ*=33.553(3)°. Diffraction data were recorded with an accumulation time of 3 min during active electrochemical operation and during the relaxation periods. The full diffractograms were recorded by merging neutron data obtained in several steps while moving the detector in 2*θ*=10° steps with an accumulation time of 10 min.

For the low‐temperature measurements, cells were fixed to a sample stick and inserted into the sample tube of a liquid‐refrigerant‐free closed cycle CCR‐type cryostat. With this specially designed rotation sample stick, the sample position could be matched to the neutron beam by a simple height adjustment at the bottom part of the sample stick. For temperature regulation of the cell, a Lakeshore temperature controller, sensors, and heater attached to the sample tube were used.

The raw data correction and reduction were performed with the StressTextureCalculator (STeCa) software.^50^ Thereafter, integral reflection intensities of the evolving LiC_12_ (002) and LiC_6_ (001) reflections were extracted by fitting them with pseudo Voigt profiles. In rare cases, outliers were detected for the integrated intensities. The outliers are in the data itself and possible reasons for these outliers are data acquisition issues at pattern edges and sporadic errors of the neutron detector. Therefore, the fitting procedure can be excluded as the source for the outliers. Outliers are marked in faded colors.

GD‐OES was used to record elemental depth profiles from the anode surface to the current collector. The GD‐OES analysis was performed using a GDA750 device (Spectruma). A radio frequency method was used while applying a 550 V discharge voltage and a gas pressure of 2 hPa. A mixture of 1 vol. % of H_2_ in Ar (6.0 purity) was used as the analysis gas. The cell opening and post‐mortem analysis was performed directly after aging of the cells to ensure representative results. To prevent reaction of the atmosphere with the anode, an airtight sample chamber was used to transfer to sample from the glove box to the GD‐OES.

## Conflict of interest

*The authors declare no conflict of interest*.

## Supporting information

As a service to our authors and readers, this journal provides supporting information supplied by the authors. Such materials are peer reviewed and may be re‐organized for online delivery, but are not copy‐edited or typeset. Technical support issues arising from supporting information (other than missing files) should be addressed to the authors.

Supplementary

## References

[1] M. Höök , X. Tang , Energy Policy 2013, 52, 797–809.

[2] F. Maroni , G. Carbonari , F. Croce , R. Tossici , F. Nobili , ChemSusChem 2017, 10, 4771–4777. 28881495 10.1002/cssc.201701431

[3] N. Delpuech , N. Dupre , P. Moreau , J.-S. Bridel , J. Gaubicher , B. Lestriez , D. Guyomard , ChemSusChem 2016, 9, 841–848. 26915951 10.1002/cssc.201501628

[4] K. P. C. Yao , J. S. Okasinski , K. Kalaga , J. D. Almer , D. P. Abraham , Adv. Energy Mater. 2019, 9, 1803380.

[5] M. N. Obrovac , V. L. Chevrier , Chem. Rev. 2014, 114, 11444–11502. 25399614 10.1021/cr500207g

[6] N. Paul , M. Wetjen , S. Busch , H. Gasteiger , R. Gilles , J. Electrochem. Soc. 2019, 166, A1051–A1054.

[7] M. Wetjen , S. Solchenbach , D. Pritzl , J. Hou , V. Tileli , H. A. Gasteiger , J. Electrochem. Soc. 2018, 165, A1503–A1514.

[8] P. Limthongkul , Y.-I. Jang , N. J. Dudney , Y.-M. Chiang , Acta Mater. 2003, 51, 1103–1113.

[9] K. Richter , T. Waldmann , M. Memm , M. Kasper , M. Wohlfahrt-Mehrens , J. Electrochem. Soc. 2018, 165, A3602–A3604.

[10] K. Richter , T. Waldmann , M. Kasper , C. Pfeifer , M. Memm , P. Axmann , M. Wohlfahrt-Mehrens , J. Phys. Chem. C 2019, 123, 18795–18803.

[11] C. Exley , J. Inorg. Biochem. 1998, 69, 139–144. 10.1016/s0162-0134(97)10012-5 9629673

[12] J. B. Quinn , T. Waldmann , K. Richter , M. Kasper , M. Wohlfahrt-Mehrens , J. Electrochem. Soc. 2018, 165, A3284–A3291.

[13] J. R. Dahn , Phys. Rev. B 1991, 44, 9170–9177. 10.1103/physrevb.44.9170 9998896

[14] N. Legrand , B. Knosp , P. Desprez , F. Lapicque , S. Raël , J. Power Sources 2014, 245, 208–216.

[15] J. Vetter , P. Novák , M. R. Wagner , C. Veit , K.-C. Möller , J. O. Besenhard , M. Winter , M. Wohlfahrt-Mehrens , C. Vogler , A. Hammouche , J. Power Sources 2005, 147, 269–281.

[16] T. Waldmann , B.-I. Hogg , M. Wohlfahrt-Mehrens , J. Power Sources 2018, 384, 107–124.

[17] S. Hein , A. Latz , Electrochim. Acta 2016, 201, 354–365.

[18] T. Waldmann , B.-I. Hogg , M. Kasper , S. Grolleau , C. G. Couceiro , K. Trad , B. P. Matadi , M. Wohlfahrt-Mehrens , J. Electrochem. Soc. 2016, 163, A1232–A1238.

[19] H.-P. Lin , D. Chua , M. Salomon , H.-C. Shiao , M. Hendrickson , E. Plichta , S. Slane , Electrochem. Solid-State Lett. 2001, 4, A71.

[20] T. Waldmann , J. B. Quinn , K. Richter , M. Kasper , A. Tost , A. Klein , M. Wohlfahrt-Mehrens , J. Electrochem. Soc. 2017, 164, A3154–A3162.

[21] V. Zinth , C. von Lüders , M. Hofmann , J. Hattendorff , I. Buchberger , S. Erhard , J. Rebelo-Kornmeier , A. Jossen , R. Gilles , J. Power Sources 2014, 271, 152–159.

[22] C. von Lüders , V. Zinth , S. V. Erhard , P. J. Osswald , M. Hofmann , R. Gilles , A. Jossen , J. Power Sources 2017, 342, 17–23.

[23] C. Uhlmann , J. Illig , M. Ender , R. Schuster , E. Ivers-Tiffée , J. Power Sources 2015, 279, 428–438.

[24] Z. P. Hu , A. Ignatiev , Phys. Rev. B 1984, 30, 4856–4859.

[25] M. T. Johnson , H. I. Starnberg , H. P. Hughes , Surf. Sci. 1986, 178, 290–299.

[26] J. C. Burns , D. A. Stevens , J. R. Dahn , J. Electrochem. Soc. 2015, 162, A959–A964.

[27] T. Weigel , F. Schipper , E. M. Erickson , F. A. Susai , B. Markovsky , D. Aurbach , ACS Energy Lett. 2019, 4, 508–516.

[28] T. D. Tran , J. H. Feikert , R. W. Pekala , K. Kinoshita , J. Appl. Electrochem. 1996, 26, 1161–1167.

[29] J. Jiang , Q. Liu , C. Zhang , W. Zhang , IEEE Trans. Ind. Electron. 2014, 61, 6844–6851.

[30] A. Klein , P. Axmann , M. Wohlfahrt-Mehrens , J. Electrochem. Soc. 2016, 163, A1936–A1940.

[31] H. Tanida , H. Yamashige , Y. Orikasa , Y. Gogyo , H. Arai , Y. Uchimoto , Z. Ogumi , J. Phys. Chem. C 2016, 120, 4739–4743.

[32] T. Numata , C. Amemiya , T. Kumeuchi , M. Shirakata , M. Yonezawa , J. Power Sources 2001, 97–98, 358–360.

[33] N. Ghanbari , T. Waldmann , M. Kasper , P. Axmann , M. Wohlfahrt-Mehrens , J. Phys. Chem. C 2016, 120, 22225–22234.

[34] D. A. Morton-Blake , J. Corish , F. Bénière , Phys. Rev. B 1988, 37, 4180–4187. 10.1103/physrevb.37.4180 9945055

[35] K. Persson , Y. Hinuma , Y. S. Meng , A. Van der Ven , G. Ceder , Phys. Rev. B 2010, 82, 125416.

[36] H. Kim , K. E. Kweon , C.-Y. Chou , J. G. Ekerdt , G. S. Hwang , J. Phys. Chem. C 2010, 114, 17942–17946.

[37] C.-Y. Chou , H. Kim , G. S. Hwang , J. Phys. Chem. C 2011, 115, 20018–20026.

[38] T. C. Bach , S. F. Schuster , E. Fleder , J. Müller , M. J. Brand , H. Lorrmann , A. Jossen , G. Sextl , J. Energy Storage 2016, 5, 212–223.

[39] M. Petzl , M. Kasper , M. A. Danzer , J. Power Sources 2015, 275, 799–807.

[40] N. Paul , J. Keil , F. M. Kindermann , S. Schebesta , O. Dolotko , M. J. Mühlbauer , L. Kraft , S. V. Erhard , A. Jossen , R. Gilles , J. Energy Storage 2018, 17, 383–394.

[41] N. Paul , J. Wandt , S. Seidlmayer , S. Schebesta , M. J. Mühlbauer , O. Dolotko , H. A. Gasteiger , R. Gilles , J. Power Sources 2017, 345, 85–96.

[42] A. Iturrondobeitia , F. Aguesse , S. Genies , T. Waldmann , M. Kasper , N. Ghanbari , M. Wohlfahrt-Mehrens , E. Bekaert , J. Phys. Chem. C 2017, 121, 21865–21876.

[43] T. Waldmann , N. Ghanbari , M. Kasper , M. Wohlfahrt-Mehrens , J. Electrochem. Soc. 2015, 162, A1500–A1505.

[44] M. Safari , C. Delacourt , J. Electrochem. Soc. 2011, 158, A1436.

[45] T. Danner , M. Singh , S. Hein , J. Kaiser , H. Hahn , A. Latz , J. Power Sources 2016, 334, 191–201.

[46] N. Ghanbari , T. Waldmann , M. Kasper , P. Axmann , M. Wohlfahrt-Mehrens , ECS Electrochem. Lett. 2015, 4, A100–A102.

[47] S. Tippmann , D. Walper , L. Balboa , B. Spier , W. G. Bessler , J. Power Sources 2014, 252, 305–316.

[48] C. Hogrefe, T. Waldmann, K. Richter, M. Wohlfahrt-Mehrens, in preparation.

[49] T. Waldmann , M. Wilka , M. Kasper , M. Fleischhammer , M. Wohlfahrt-Mehrens , J. Power Sources 2014, 262, 129–135.

[50] C. Randau , U. Garbe , H.-G. Brokmeier , J. Appl. Crystallogr. 2011, 44, 641–646.

