# Supplementary material for: Low‐Temperature Charging and Aging Mechanisms of Si/C Composite Anodes in Li‐Ion Batteries: An Operando Neutron Scattering Study
Source: ChemSusChem. 2019 Dec 27;13(3):529–38. doi: 10.1002/cssc.201903139 (PMC7028078; doi:10.1002/cssc.201903139)
Supplement: Supplementary file 1 — Supplementary [file CSSC-13-529-s001.pdf]

CHEMISTRY & SUSTAINABILITY
CHEMSUSCHEM
ENERGY & MATERIALS

Supporting Information

Low-Temperature Charging and Aging Mechanisms of Si/C Composite Anodes in Li-Ion Batteries: An Operando Neutron Scattering Study

Karsten Richter,[a] Thomas Waldmann,*[a] Neelima Paul,[b] Nicola Jobst,[a] Rares-George Scurtu,[a] Michael Hofmann,[b] Ralph Gilles,[b] and Margret Wohlfahrt-Mehrens[a]

cssc_201903139_sm_miscellaneous_information.pdf

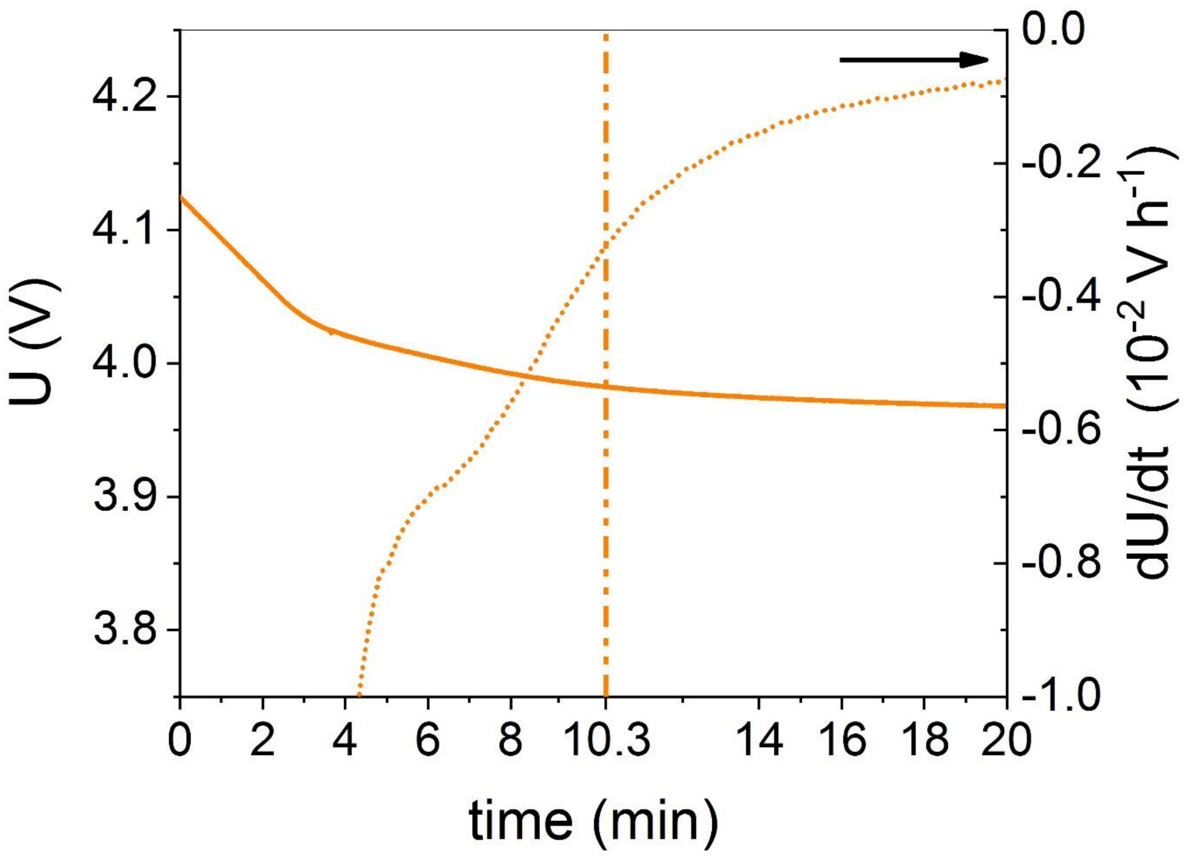

FigureS1. Evolution of the cell voltage (left axis) and differential voltage curve (right axis) after charging with 0.5 C at (-21 ± 2) °C.

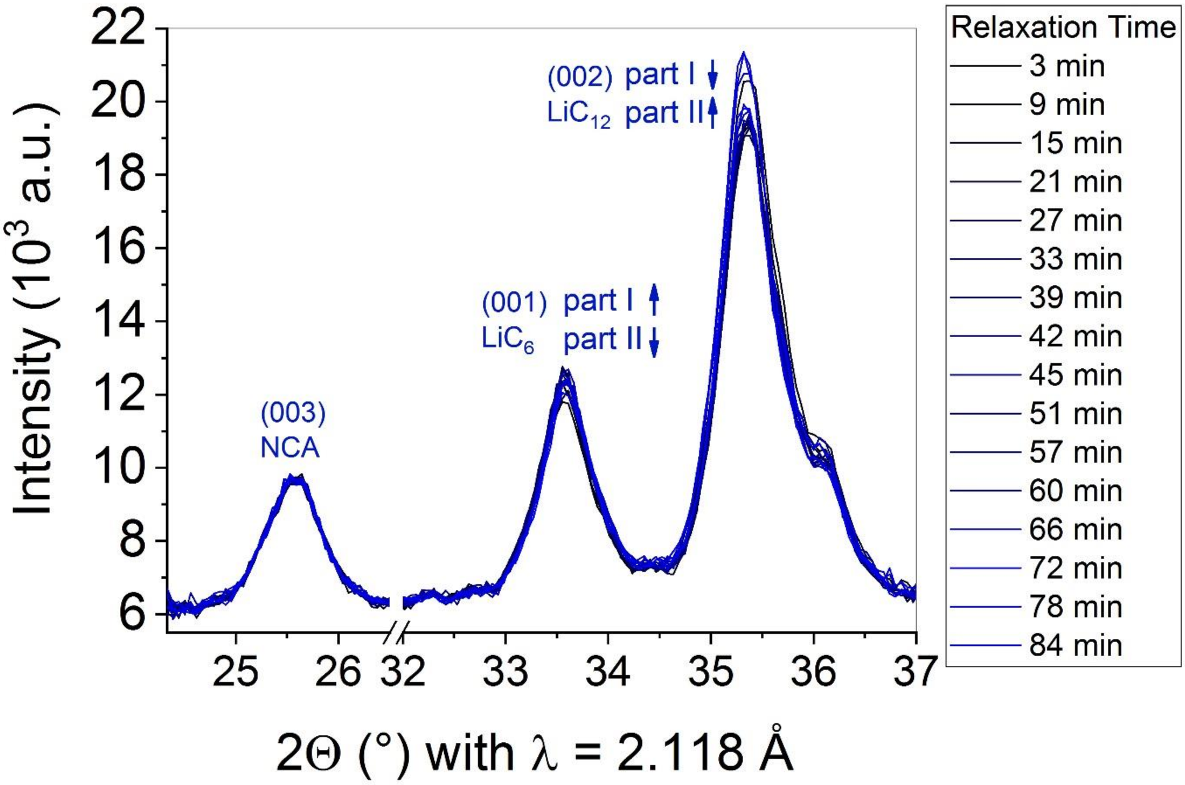

FigureS2. Time dependent evolution of the NCA(003), LiC_6(001) and the LiC_{12}(002) reflections during the relaxation period after charging. Exemplarily shown for the fresh cell after charging with 0.75 C at -18 °C.

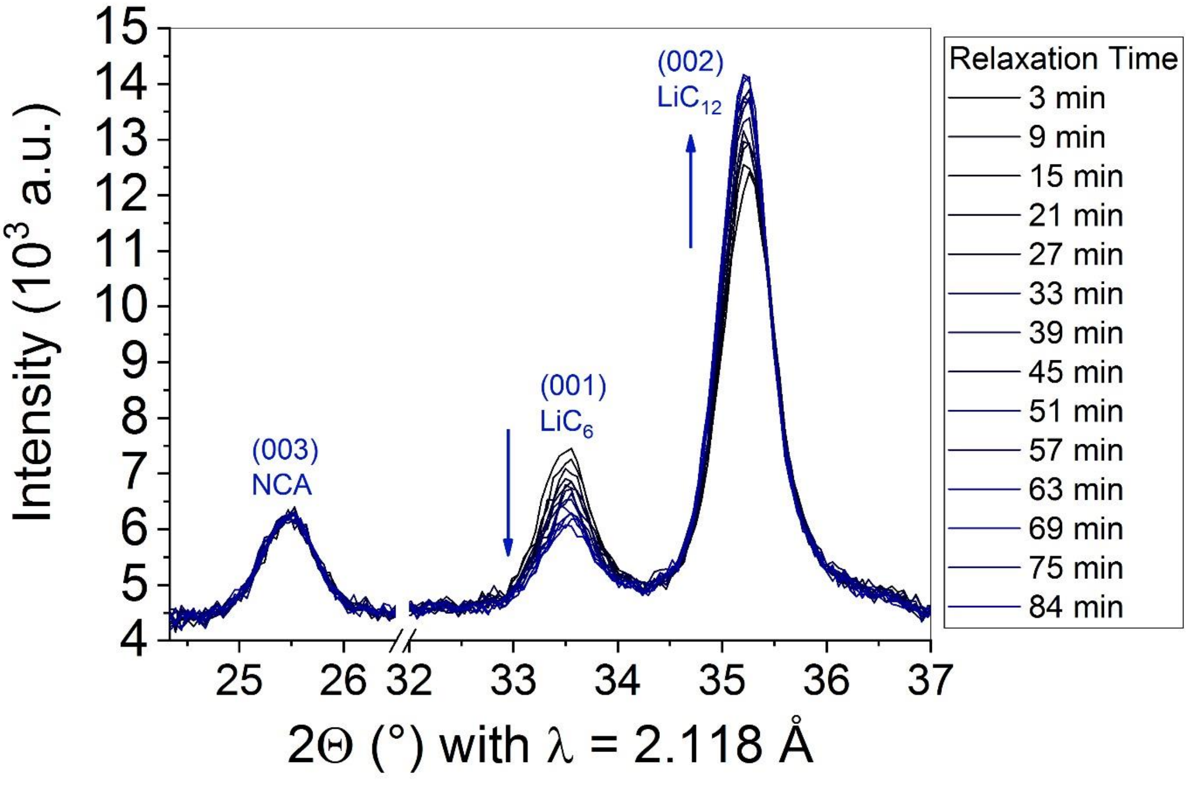

FigureS3. Time dependent evolution of the NCA(003), LiC_6(001) and the LiC_{12}(002) reflections during the relaxation period after charging. Exemplarily shown for the aged cell after charging with 0.75 C at -10 °C.
